# RNAtips: analysis of temperature-induced changes of RNA secondary structure

**DOI:** 10.1093/nar/gkt486

**Published:** 2013-06-12

**Authors:** Andrey Chursov, Sebastian J. Kopetzky, Gennady Bocharov, Dmitrij Frishman, Alexander Shneider

**Affiliations:** ^1^Department of Genome Oriented Bioinformatics, Technische Universität München, Wissenschaftzentrum Weihenstephan, Maximus-von-Imhof-Forum 3, D-85354 Freising, Germany, ^2^Institute of Numerical Mathematics, Russian Academy of Sciences, Gubkina str. 8, 119333 Moscow, Russia, ^3^Helmholtz Center Munich-German Research Center for Environmental Health (GmbH), Institute of Bioinformatics and Systems Biology, Ingolstädter Landstraße 1, D-85764 Neuherberg, Germany and ^4^Cure Lab, Inc., 43 Rybury Hillway, Needham, MA 02492, USA

## Abstract

Although multiple biological phenomena are related to temperature (e.g. elevation of body temperature due to an illness, adaptation to environmental temperature conditions, biology of coldblooded versus warm-blooded organisms), the molecular mechanisms of these processes remain to be understood. Perturbations of secondary RNA structures may play an important role in an organism’s reaction to temperature change—in all organisms from viruses and bacteria to humans. Here, we present RNAtips (temperature-induced perturbation of structure) web server, which can be used to predict regions of RNA secondary structures that are likely to undergo structural alterations prompted by temperature change. The server can also be used to: (i) detect those regions in two homologous RNA sequences that undergo different structural perturbations due to temperature change and (ii) test whether these differences are specific to the particular nucleotide substitutions distinguishing the sequences. The RNAtips web server is freely accessible without any login requirement at http://rnatips.org.

## INTRODUCTION

Structural perturbations in RNA molecules induced by temperature change may have important biological implications. For instance, the stability of mRNA structural elements in 5′-untranslated regions correlates with the translation rate in *Saccharomyces cerevisiae* (1). Another example is the temperature-sensitivity of cold-adapted influenza vaccine strains. For decades, it was a conundrum why wild-type influenza strains react differently to elevated temperature than their cold-adapted temperature-sensitive counterparts. Recently, it has been demonstrated that this difference in temperature sensitivity may be due to the difference in temperature-induced perturbations in mRNA secondary structures (2). Perhaps, the most widely known example is RNA thermometers, which at a particular temperature alter their structure, and regulate translation of heat-shock, cold-shock and virulence genes (3–8). Usually, RNA thermometers are located in 5′-untranslated regions, and their structures melt at an elevated temperature thereby permitting ribosomes to initiate the translation process.

There are several experimental approaches to measuring the melting temperature of an RNA structure (9), including ultraviolet absorbance (10,11), ﬂuorescence-based techniques (12,13) and thermal gradient electrophoresis (14–16). Recently, temperature stability of RNA structural elements was assessed on a genome-wide basis (17). The Parallel Analysis of RNA Structures with Temperature Elevation technique was applied to the yeast transcriptome, and relative melting temperatures for RNA structures were obtained by probing RNA structures at different temperatures from 23 to 75°C. As a result of this assessment, thousands of potential RNA thermometers and highly temperature-stable structures were identified.

Temperature-induced perturbations of RNA structures may play crucial, and yet unknown, biological role(s) in a variety of processes. Elevation of body temperature is the most common symptom of many illnesses. The effects of elevated body temperature on RNA structures both in pathogens and their hosts are still unknown, although they may constitute a defense mechanism. Additionally, it would be interesting to assess whether RNA temperature sensitivity plays an evolutionary role in organism adaptation to different climate zones, as well as to seasonal and day–night temperature change. The latter question is especially important owing to global climate change. Is temperature sensitivity of RNA structures in bacteria living in geysers different from that of bacteria living at negative temperatures? Do RNA structures from warm-blooded organisms react to the temperature change similarly to their counterparts in cold-blooded animals? These and many other questions could not be systematically addressed, however, as (to the best of our knowledge) there is no convenient instrument to identify and compare temperature-sensitive regions of RNA molecules.

To close this gap, RNAtips (temperature-induced perturbation of structure) web server has been developed. For a single RNA sequence, RNAtips identifies (i) those nucleotides for which temperature change causes appreciable alteration of the probability to form Watson–Crick (W–C) pairs and (ii) clusters of such temperature-sensitive nucleotides. If the research goal is to compare two RNA sequences and identify whether they react differently to a temperature change, the locations of temperature-sensitive clusters within the two RNAs are compared. If the two sequences are homologs with a limited number of base substitutions, an analysis can be performed to demonstrate whether the difference in location of the temperature-sensitive clusters between the two sequences is specific to these particular nucleotide substitutions, or if it could be achieved with the same number of random mutations (synonymous and/or non-synonymous).

## METHOD SUMMARY

The methodology implemented in RNAtips web server for assessing such impacts of temperature change was previously described and published by Chursov *et al.* (2). In short, each nucleotide within an RNA sequence has a probability of being paired via W–C bonds. This probability is temperature dependent; therefore, temperature changes influence the probability of forming W–C pairs for each and every nucleotide. However, some nucleotides change their pairing probabilities to a much greater extent than others. Moreover, these highly temperature-sensitive nucleotides may not be evenly distributed along the RNA sequence but rather form distinct clusters (2). Thus, the first task performed by the RNAtips web server is identification of those positions, which are prone through temperature elevation to significantly change their probability of being paired. This task is performed through the following steps. Step 1: Probabilities of nucleotides to be coupled within a double-stranded conformation are assessed at each temperature within the given range by using the RNAfold tool of the ViennaRNA package (18). Step 2: For each nucleotide, RNAtips calculates the difference in probability for it to be in a paired state at the lower temperature and at the higher one. These differences are calculated for the entire temperature range (t_1_ : t_2_) [i.e. for (t_1_ + 1) − t_1_, … , t_2_ − t_1_] and then combined into one data set. For example, if the temperature range is set to 32–39°C and the length of the sequence is 1000, then the changes of probabilities are considered for 33°C compared with 32°C, 34°C compared with 32°C, … , 39°C compared with 32°C, and the final data set would contain 7000 values. Step 3: The server identifies the most temperature-sensitive positions. For this purpose, the server selects those values (and their corresponding nucleotides) from the data set generated in Step 2, which are distant from the mean by more than three standard deviations (the default value can be changed by the user). The server then considers these positions to be the most temperature-sensitive, and they are then mapped on the original sequence. Furthermore, clusters of significantly changing positions are then identified by applying the density-based spatial clustering of applications with noise (DBSCAN) algorithm to the locations of such positions. The server default action is to apply the cluster analysis algorithm only to the highest temperature differences t_2_-t_1_, (32–39°C in the previous example) (19,20).

It may be important to assess whether structures of RNA molecules sharing sequence similarity react (dis)similarly to temperature change. For simplicity of explanation, assume that one RNA sequence was derived from another sequence via some mutations. Then, the second task, which can be performed by RNAtips server, is to identify whether structures of two homologous RNA sequences react differently to the temperature change and, if they do, whether this difference can be attributed to the specific mutations separating the two homologous sequences. Thus, if a user inputs two sequences, RNAtips identifies clusters of temperature-sensitive positions, which could be either common for both sequences or uniquely present in only one of the two RNA molecules. If the clusters of temperature-sensitive positions are not identical for the two sequences, the server offers statistical analysis identifying whether the difference in temperature sensitivity is specific to the particular nucleotide substitutions naturally differentiating the sequences or whether any set of mutations comparable in size could lead to the same difference.

Therefore, assume that N nucleotide substitutions differentiate sequence A from sequence B. The server generates a data set of derivative sequences for A introducing N substitutions into each derivative sequence. There are two different methods of introducing random substitutions into a sequence(s) depending on whether the sequence(s) is(are) non-coding or coding. If A is a coding sequence (default), mutants will be generated by introducing synonymous mutations only. If A is a non-coding sequence, the user should mark a checkbox: ‘The input sequence(s) is(are) non-coding’. In this case, *in silico* mutations will be introduced at random positions mimicking frequencies of nucleotide substitutions naturally occurring between A and B (e.g. if 25% of nucleotide substitutions between A and B are T->C, then T->C substitutions will be introduced in 25% of random *in silico* mutations). For each computer-generated sequence, the server will calculate its clusters of the temperature-sensitive positions as described earlier in the text. If sequence B has a sequence-specific cluster of temperature-sensitive positions not present in A, some of *in silico* derivatives of A may possess clusters overlapping with the sequence-specific cluster observed in B. Let us assume that 1% or less of computer-generated sequences possess such clusters overlapping with the sequence-specific cluster in B. This means that 99% of random mutation sets did not lead to the appearance of this sequence-specific cluster of temperature sensitivity specific for sequence B, but not for A. Thus, one can conclude that the RNA structure of sequence B reacts to the temperature change differently than the structure of sequence A because it possesses a specific set of mutations as opposed to just N non-specific mutations. The RNAtips server performs a statistical analysis calculating a *P*-value for every sequence-specific cluster by performing a one-sided binomial test. For sequence-specific clusters occurring in the first sequence but not in the second one, the null hypothesis (H_0_) is that the probability to observe this cluster is <95%. Consequently, a small *P*-value shows that the cluster is unlikely to disappear in the second sequence by chance. For sequence-specific clusters occurring in the second sequence but not in the first one, the null hypothesis (H_0_) is that the probability to observe this cluster amongst the mutants generated *in silico* is ≥5%. Therefore, a small *P*-value shows that the cluster is unlikely to appear in the second sequence by chance.

## WEB SERVER

### Input data

The input for RNAtips consists of either one or two RNA sequences of the same length that should be provided in FASTA-format (the header can be omitted). The sequences can either be uploaded as text files (each file may contain only one sequence), or the sequences may be directly pasted into an input field. The sequences may contain the characters A, C, G, U and T (for further computations, all Thymidines will be replaced with Uracils automatically). The maximal length of the sequences is limited to 9999 nt. To see an example of possible input sequences, the user can click on the ‘sample’ link on the Start page. Influenza strains A/Leningrad/134/57 and its cold-adapted temperature-sensitive mutant A/Leningrad/134/47/57 are used as sample sequences.

Additionally, a user has to specify two temperatures t_1_ and t_2_ (in °C) to define the temperature range (t_1_ : t_2_) for which the RNA structural perturbation should be calculated (the default range is 32–39°C). t_2_ is the temperature for which the actual cluster identification will be performed. The minimal allowed temperature is 0°C, and the maximal allowed temperature is 99°C. Furthermore, the maximal allowed temperature difference (i.e. t_2_ - t_1_ + 1) is restricted to 20°C (e.g. 30–49°C).

If a user inputs two sequences, two options are available. The default option is to perform a statistical analysis of the sequence-specific clusters identified in each of the two sequences and to test whether these sequence-specific clusters result from the particular set of mutations distinguishing the sequences. However, this analysis takes some time and may be unnecessary for the particular user. In this case, the user can choose the checkbox for the ‘Don't Create a Mutant Dataset’ option (this option is only relevant for two input sequences).

An advanced user can deviate from this default setup and input his parameters of choice. It was described in the ‘Method Summary’ section that the statistical threshold for identifying significantly changing positions is 3.0 standard deviations. However, in a custom calculation, a user may also choose a threshold level other than 3.0. The next two advanced parameters, ε and MinPts, are both parameters for the clustering algorithm called DBSCAN. As the first step, the algorithm randomly selects one significantly changing position. MinPts specifies the minimal number of significantly changing positions in a cluster. ε specifies the distance from the chosen nucleotide. If the number of significant positions specified by MinPts is located within distance ε, the sequence segment is then considered part of a cluster. The default values for ε and MinPts are 11 and 5, respectively.

Finally, a user can then select checkbox options: ‘Don’t allow GU pairs at the end of helices’ and/or ‘Don’t allow GU pairs’. These selections instruct the server whether GU pairs should be considered in calculations when the probabilities of nucleotides to be in a double-stranded confirmation at any given temperature are calculated. These two checkboxes are converted into the –noCloseGU and –noGU parameters of RNAfold during the calculations of probabilities of nucleotides to be coupled.

### Server output

**Figure 1.**
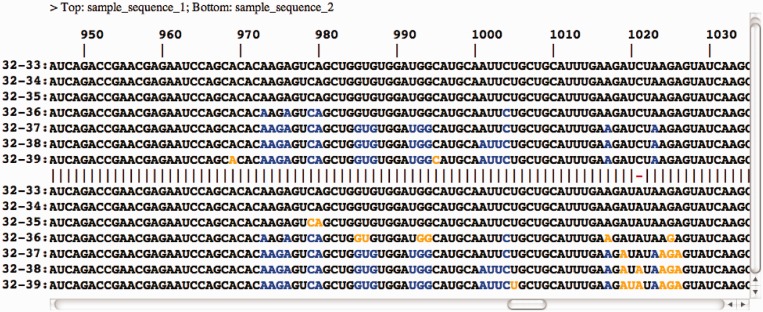
Comparison of significantly changing positions between two RNAs. Top and bottom halves of the figure demonstrate influenza strains A/Leningrad/134/57 and its cold-adapted temperature-sensitive mutant A/Leningrad/134/47/57, respectively. Each row corresponds to a particular temperature difference. Positions in which base pairing probabilities significantly change with temperature elevation in both sequences and those where these changes only affect one of the sequences are marked blue and orange, respectively. Position numbers are indicated at the top of the alignment.

At the top of the results page from RNAtips, the HTML output provides colored visual representation of identified temperature-sensitive positions (Figure 1). The left column contains values for each temperature within the temperature range (t_1_ : t_2_). The right column presents the input sequence with those nucleotides—which are the most temperature-sensitive at this temperature—marked in either blue or orange color. The header line presents the FASTA header of the sequence(s). Position numbers are indicated under the header line. In the case of two input sequences, a line between the results for both sequences indicated matching positions with ‘**|**’ and mismatches (mutations) with ‘**-**’. Additionally, significant positions that are sequence specific to one of the two sequences only are displayed in orange color. Positions that change their probabilities to be paired significantly in both sequences are displayed in blue color. If only one sequence is used as an input, then all positions demonstrating the highest potency to change their likelihood of forming W–C bonds are displayed in orange color. In addition, the HTML output demonstrates the temperature initiating a perturbation of the RNA structure.

All tables and figures presenting more detailed results are shown in the lower part of the page and described in the following paragraphs. The first table displays general information on identified significant positions and clusters. For every input sequence, it has the following fields: ‘Sequence’ (shows the ID of the input sequence); ‘#significant positions/total length’ (the number of significantly changing positions and the total length of the input sequence); ‘signif. pos. < 0/signif. pos. > 0’ [the numbers of significantly changing positions that decrease (or increase) their probability to be paired with temperature elevation]; ‘Number of clusters’ (the total number of identified clusters of significantly changing positions); ‘Avg. cluster density’ (the average density of significantly changing positions in the identified clusters); and ‘Avg. cluster length’ (the average length of the identified clusters). The probability difference values are calculated by subtracting the value at the highest temperature from the value at the lowest temperature (p_39°C_ - p_32°C_ for the previous example). Cluster density is calculated as the number of significantly changing positions in a cluster divided by the total length of the cluster.

**Figure 2.**
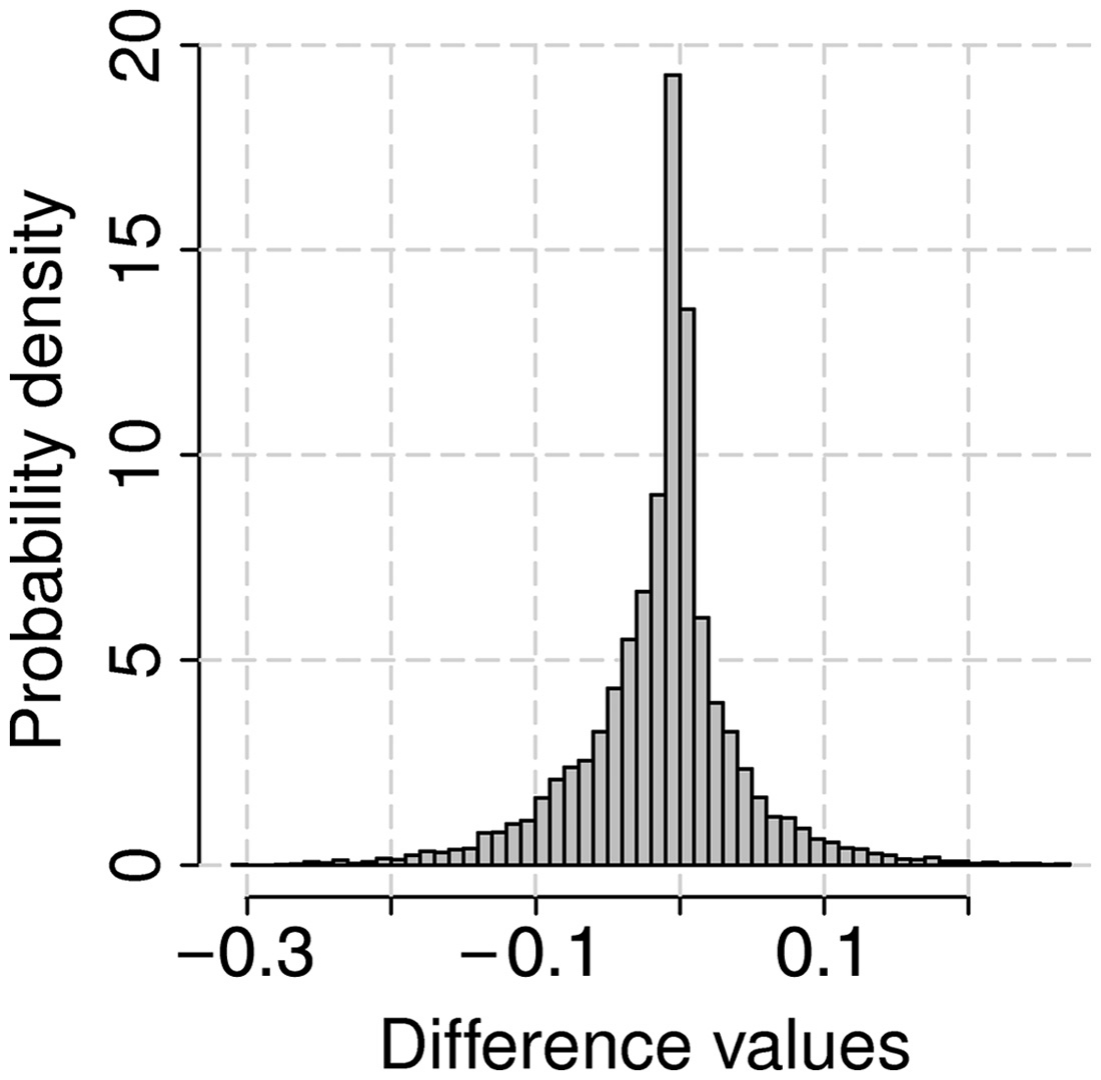
The histogram of differences in probability values of nucleotides to be in a double-stranded conformation for mRNA of nucleoprotein (NP) of influenza strain A/Leningrad/134/57 on temperature change between 32 and 39°C. The probability values of nucleotides to be paired for 32°C were subtracted from the probability values for every temperature from 33 to 39°C. All the differences were combined into one data set.

If a sequence contains 1000 nt and the temperature changes from 32 to 39°C, there are 7000 values reflecting how much each nucleotide would change its probability to form W–C couples when the temperature increases from 32 to 33°C, from 32 to 34°C, … , from 32 to 39°C. The output for this example would contain a histogram over all these 7000 data points (Figure 2). These histograms are used to identify the most temperature-sensitive positions (by default, further than 3 standard deviations away from the mean value). Overall, a histogram contains (t_2_ - t_1_)*(sequence length) values. For every input sequence, one histogram is presented at the output page. Thus, if two closely related sequences were used to compare their temperature sensitivity, the output would possess two histograms.

**Figure 3.**
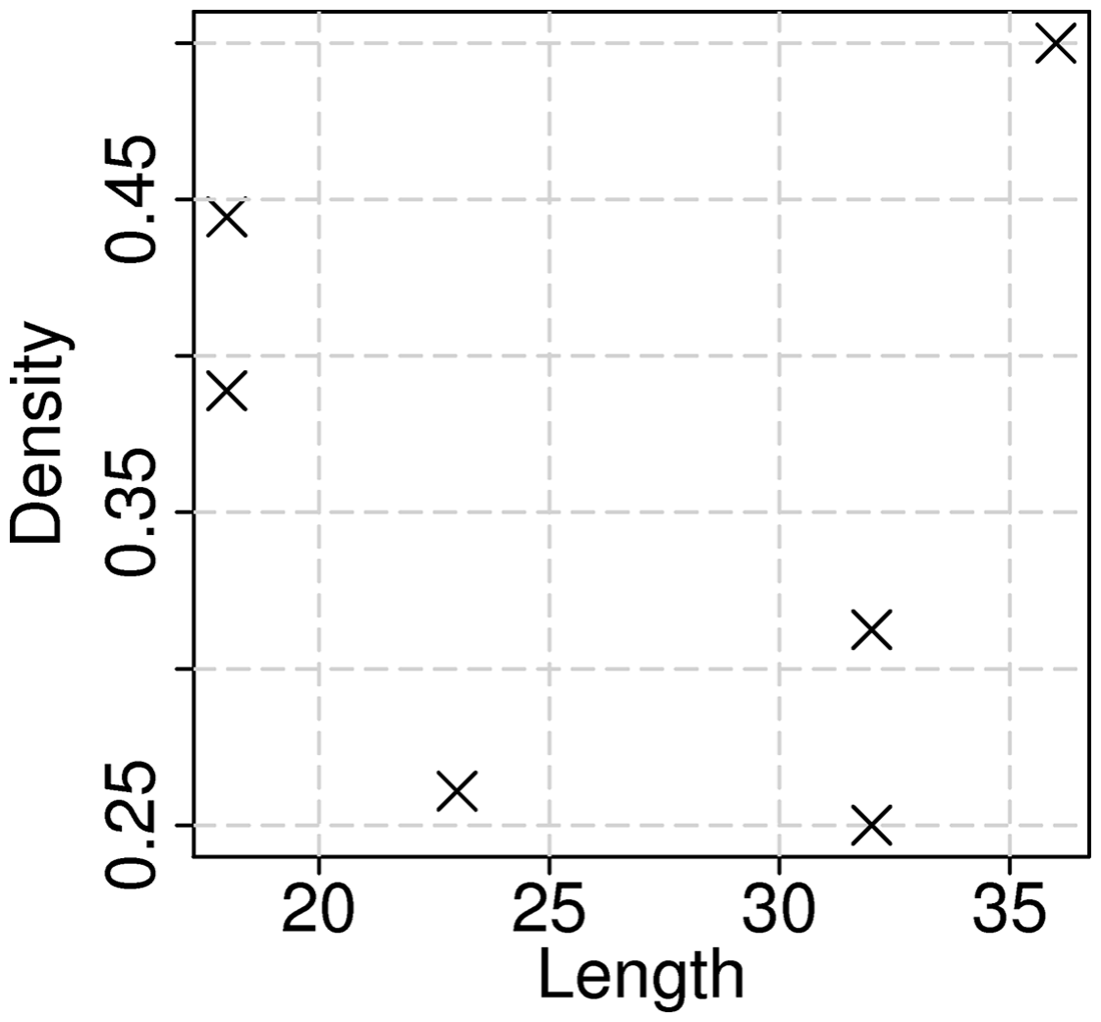
Density of significantly changing positions in determined clusters versus length of those clusters. Clusters were identified for mRNA of nucleoprotein (NP) of influenza strain A/Leningrad/134/57 by applying default parameters of the web server.

The exact location (start and end positions) of the identified clusters (if any) is shown in the following table. The accompanying output figure shows the relationship between the length and density of the clusters (Figure 3). In this figure, each point represents one cluster. The cluster density is plotted versus the cluster length. Several clusters can have the same properties, and in such a case, the corresponding points will overlap. Therefore, the total number of apparent points can be different from the total number of clusters. Such tables and figures are presented for every sequence in which clusters of the most temperature-sensitive positions were identified. Otherwise, the web server directly indicates that no clusters were identified for a particular sequence.

**Figure 4.**
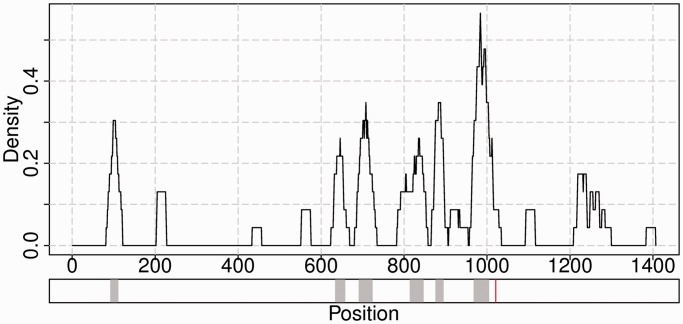
The upper panel demonstrates a density plot of significantly changing positions along the input sequence. A sliding window of size 2*ε + 1 is moved in steps of 1 position over the sequence with the highest temperature difference t_2_ − t_1_. The percentage of significantly changing positions in the window is calculated for each possible starting position. The bottom panel shows location of clusters of significantly changing positions identified by the DBSCAN algorithm depicted with gray color, and mutation is depicted with red vertical line. The depicted mutation corresponds to the nucleotide difference between influenza strains A/Leningrad/134/57 and its cold-adapted temperature-sensitive mutant A/Leningrad/134/47/57.

For every input sequence, the following figure demonstrates density of the most temperature-sensitive positions over the whole RNA sequence together with localization of clusters and localization of nucleotide substitutions (if any) (Figure 4). The upper part of this figure is created by moving a sliding window of size 2*ε + 1 over the corresponding sequence and determining the density of significantly changing positions within it. The lower part shows the localization of clusters and mutation sites on the sequence.

As described earlier in the text, if two homologous RNA sequences constitute an input, one of the sequences may possess clusters of temperature-sensitive nucleotides, which are not present in the other RNA molecule (i.e. clusters that can be found for the given DBSCAN parameters in one RNA, and they do not overlap with any clusters from the other RNA). Appearance of these sequence-specific clusters may be a specific consequence of the particular nucleotide substitutions differentiating the RNAs. Alternatively, the clusters could result from a high number of non-specific mutations. Results of the statistical analysis presented in the last table (if conducted) demonstrate whether a sequence-specific temperature-sensitive cluster observed in one RNA but not in another is due to specific nucleotide substitutions taking place in the sequences. In other words, these data demonstrate whether such a specific difference between the two RNAs can be achieved by introducing the same number of random mutations. The server generates a data set of *in silico* mutants for the first RNA as described in the ‘Method Summary’ section. Some of these *in silico* mutants may possess temperature-sensitive clusters, which are not present in the original RNA sequence. The table shows positions of sequence-specific clusters observed in the RNA sequence, the frequency for each sequence-specific cluster to be overlapping with a cluster in the computer-generated mutants (at least, by one position), the *P*-value and 95% confidence interval calculated from the binomial test for each sequence-specific cluster to be a result of a random mutation set introduced into the original RNA.

All figures and additional information can be downloaded by RNAtips users. The results page enables a user to download a zip-file of all sequences of the *in silico* mutants (if generated). Results of every job will be stored on the server for at least 3 days. Every submitted job receives a unique URL and a user can browse the results during this period.

### Implementation

RNAtips web server has a user-friendly interface and runs under the Linux operating system. The server’s back-end, including the core part of computations as well as implementation of the DBSCAN algorithm, is written in Python. Statistical tests and generation of plots are implemented in R programming language. Calculation of probabilities of nucleotides to be paired in a double-stranded conformation is performed by using the RNAfold tool of the ViennaRNA package. The front-end part of the web server is implemented in HTML markup language with dynamic parts written in JavaScript programming language. A MySQL database is used to store the input parameters and results of the computations. The server contains a help page with detailed explanation of its functionality.

## DISCUSSION

Before this presentation of RNAtips web server, researchers did not have a simple and feasible way to evaluate the affect of temperature change on secondary RNA structure. RNAtips is based on the analysis proposed and described by Chursov *et al.* (2). The name RNAtips stands for ‘temperature-induced perturbation of structure’. This server can be used to analyze localization of temperature-induced changes in the secondary structures of RNA and to compare such changes between two sequences of the same length. There are at least three advantages of using RNAtips web server instead of simply calculating the probabilities of nucleotides to be paired at two different temperatures and then comparing those probabilities. First, RNAtips deciphers those nucleotides within the RNA sequence, which change the most in their probability to form W–C bonds in response to a given temperature change. The web server demonstrates clusters of these positions within a sequence, which constitute the most temperature-sensitive structural regions. The second major benefit of RNAtips is the tool it provides to compare whether RNA structures of two closely related sequences would react (dis)similarly to a temperature change. If two RNA molecules possess different clusters of temperature-sensitive positions, their RNA structures react to the temperature change differently. Furthermore, if two RNA sequences are distinct in some nucleotide substitutions, RNAtips can be used to analyze whether either the difference in temperature sensitive clusters is specific to these particular nucleotide substitutions or whether it was likely to be caused by a similar number of non-specific nucleotide substitutions. Finally, the top RNAtips’ results page is an HTML output that presents the temperature initiating a perturbation of secondary structure in a particular temperature-sensitive region. To the best of our knowledge, no other server provides these options. RNAtips web server can be applied to a broad spectrum of research topics such as drug development, molecular diagnostic and disease prognosis, evolutionary mechanisms, ecology, investigation of climate change effects and many more. In addition, currently, we are preparing a downloadable version of the source code for local usage.

## FUNDING

DFG International Research Training Group ‘Regulation and Evolution of Cellular Systems’ [GRK 1563]; Russian Foundation for Basic Research [RFBR 09-04-92742]. Funding for open access charge: DFG International Research Training Group ‘Regulation and Evolution of Cellular Systems' [GRK 1563].

*Conflict of interest statement*. None declared.
